# Integrated Analysis of microRNA Expression, Lymphocyte Counts, and Metabolic Parameters in Obese Individuals

**DOI:** 10.3390/ijms27177663

**Published:** 2026-08-27

**Authors:** Kürşat Kargün

**Affiliations:** Autopsy Program, Department of Medical Services and Techniques, Vocational School of Health Services, Fırat University, Elazığ 23119, Türkiye; kkargun@firat.edu.tr

**Keywords:** obesity, microRNA, lymphocyte, inflammation, lipid metabolism

## Abstract

Obesity is a complex metabolic disorder characterized by chronic inflammation and immune system dysfunction. This study aimed to investigate hematological and metabolic parameters, as well as microRNA (miRNA) expression profiles associated with lymphocyte alterations, in order to elucidate obesity-specific immune–metabolic interactions. A total of 50 individuals with obesity and 25 healthy controls were included in the study. miRNA expression levels were determined using the Fluidigm BioMark RT-PCR system. The obtained data were analyzed in relation to lymphocyte count, lipid profile parameters including total cholesterol, triglycerides, LDL-C, HDL-C, and body mass index (BMI). In the analysis of lymphocyte-associated miRNA expression, hsa-miR-223-3p and hsa-miR-199a-3p expression levels were significantly decreased in individuals with obesity compared with the control group (*p* < 0.0045 and *p* < 0.0063, respectively), whereas hsa-miR-155-3p expression was significantly increased (*p* < 0.0484). Analyses based on BMI revealed significant associations with the expression levels of hsa-miR-199a-3p and hsa-miR-143-3p (*p* < 0.0321 and *p* < 0.0116, respectively). In addition, significant positive associations were detected between triglyceride levels and the expression of hsa-miR-424-3p and hsa-miR-375-3p (*p* < 0.0419 and *p* < 0.0302, respectively). Alterations in miRNA expression associated with lymphocyte parameters may represent an important component of obesity pathophysiology. These findings suggest that miRNAs may serve as potential biomarkers and therapeutic targets in obesity.

## 1. Introduction

Obesity is a major public health problem with an increasing prevalence worldwide and is strongly associated with cardiovascular diseases, type 2 diabetes, and various inflammatory disorders [1]. Individuals with a body mass index (BMI) of ≥30 kg/m^2^ are classified as obese, and dyslipidemia, particularly elevated LDL cholesterol (LDL-C), reduced HDL cholesterol (HDL-C), and increased triglyceride levels, is frequently observed in these individuals [2]. Genetic, environmental, and epigenetic factors are known to act together in the pathogenesis of obesity. Genetic factors are particularly prominent in cases of early-onset and severe obesity [3], and obesity is classified into monogenic [4], syndromic, and polygenic forms [5]. Polygenic obesity results from the interaction between multiple genetic variations and environmental factors [6,7,8]. Epigenetic mechanisms play an important role in the development of obesity by regulating the relationship between environmental influences and gene expression [9,10]. Among these mechanisms, microRNAs (miRNAs) have recently attracted attention as regulatory molecules involved in obesity-related biological processes [11]. MicroRNAs (miRNAs) are approximately 22-nucleotide-long non-coding RNA molecules that regulate gene expression at the post-transcriptional level by binding to the 3′ untranslated regions (3′-UTRs) of target mRNAs [12]. These molecules are involved in lipid metabolism, inflammation, and immune response processes, and exert regulatory effects on lymphocyte activation and cytokine production [13]. Obesity is not merely a disorder of energy imbalance but a chronic metabolic disease characterized by complex alterations in immune function, lipid metabolism, and gene expression. Expansion of adipose tissue enhances the release of pro-inflammatory cytokines, thereby promoting systemic low-grade chronic inflammation, in which immune cells, particularly lymphocytes, play a central role [14,15]. It has been shown that circulating lymphocyte counts are increased in individuals with obesity and that this increase is associated with inflammation, dyslipidemia, and insulin resistance [16]. Numerous miRNAs exhibiting tissue-specific and temporal expression patterns have been identified in human cells [17,18,19,20]. These molecules regulate a substantial proportion of gene expression and play broad roles in both physiological and pathological processes [21,22,23,24]. The ability of a single miRNA to regulate multiple target genes allows alterations in these molecules to contribute to the pathogenesis of various diseases [25,26,27,28,29]. Lymphocytes are not merely effector cells of the inflammatory response but also important components of molecular mechanisms involved in gene regulation [30]. In this context, lymphocyte-expressed miRNAs have emerged as critical regulators linking immune responses to metabolic processes. Moreover, miRNAs have been shown to circulate between different tissues through the bloodstream and to contribute to intercellular signaling [31]. In the present study, the term “lymphocyte-associated miRNAs” refers to circulating miRNAs showing differential expression according to peripheral blood lymphocyte counts rather than miRNAs originating specifically from lymphocytes.

Although the relationship of miRNAs with obesity and metabolic parameters has been extensively investigated, studies that integratively evaluate lymphocyte parameters and miRNA expression profiles together with metabolic indicators remain limited. Yet, lymphocytes play an important role in the inflammatory and metabolic processes accompanying obesity, both as a source and a target of miRNA expression. Therefore, this study aimed to identify miRNA expression profiles associated with lymphocyte parameters in individuals with obesity and to evaluate the relationships of these profiles with metabolic indicators. In this context, the study sought to reveal potential miRNA profiles associated with the immune and metabolic alterations accompanying obesity. The aim of this study was to investigate the relationship between circulating miRNA expression profiles obtained from whole blood, peripheral blood lymphocyte counts, and metabolic parameters in obese individuals.

## 2. Results

### 2.1. Demographic and Biochemical Findings

Demographic, biochemical, and hematological parameters were compared between the obese group (n = 50) and the healthy control group (n = 25) included in the study (Table 1).

Body weight and body mass index (BMI) values were significantly higher in the obese group than in the control group (*p* < 0.0001 for both). No significant difference was observed between the groups in terms of height (*p* = 0.7765).

In the lipid profile analysis, LDL-C and total cholesterol levels were significantly higher in individuals with obesity compared with the control group (*p* = 0.0253 and *p* < 0.0001, respectively). Triglyceride levels were also significantly increased in the obese group (*p* = 0.0144). No significant difference was found between the groups in HDL-C levels (*p* = 0.1756).

When hematological parameters were evaluated, lymphocyte counts were found to be significantly higher in individuals with obesity than in the control group (*p* = 0.0279). However, no significant differences were observed between the groups in terms of hemoglobin, neutrophil, monocyte, and platelet values (*p* > 0.05).

### 2.2. miRNA Expression Findings

In the lymphocyte-associated miRNA expression analysis, hsa-miR-223-3p and hsa-miR-199a-3p expression levels were significantly decreased in individuals with obesity compared with the control group (fold regulation: −2.47, *p* = 0.0045; and fold regulation: −2.76, *p* = 0.0063, respectively). In contrast, hsa-miR-155-3p expression was significantly increased in the obese group (fold regulation: 2.48, *p* = 0.0484) (Table 2).

### 2.3. miRNA Findings According to BMI

In group comparisons based on BMI values, statistically significant differences were observed in the expression levels of hsa-miR-199a-3p and hsa-miR-143-3p (*p* = 0.0321 and *p* = 0.0116, respectively) (Table 3).

### 2.4. Triglyceride-Associated miRNA Findings

In analyses performed according to triglyceride levels, significant differences were detected in the expression levels of hsa-miR-424-3p and hsa-miR-375-3p (*p* = 0.0419 and *p* = 0.0302, respectively) (Table 4).

Although all miRNAs included in the panel were analyzed, only those showing statistically significant differences are listed. The use of a comprehensive miRNA panel in this study enabled the evaluation not only of individual microRNA alterations in obesity but also of system-level molecular reprogramming. This approach allowed the integrated assessment of inflammation, metabolism, and immune response-related processes.

Negative fold regulation values indicate downregulation of the corresponding miRNA, whereas positive values indicate upregulation.

The miRNAs associated with increased BMI were considered to be predominantly involved in metabolic regulation and adipogenesis-related processes.

miRNA alterations associated with triglyceride levels may influence lipid metabolism and energy homeostasis.

**Figure 1:**
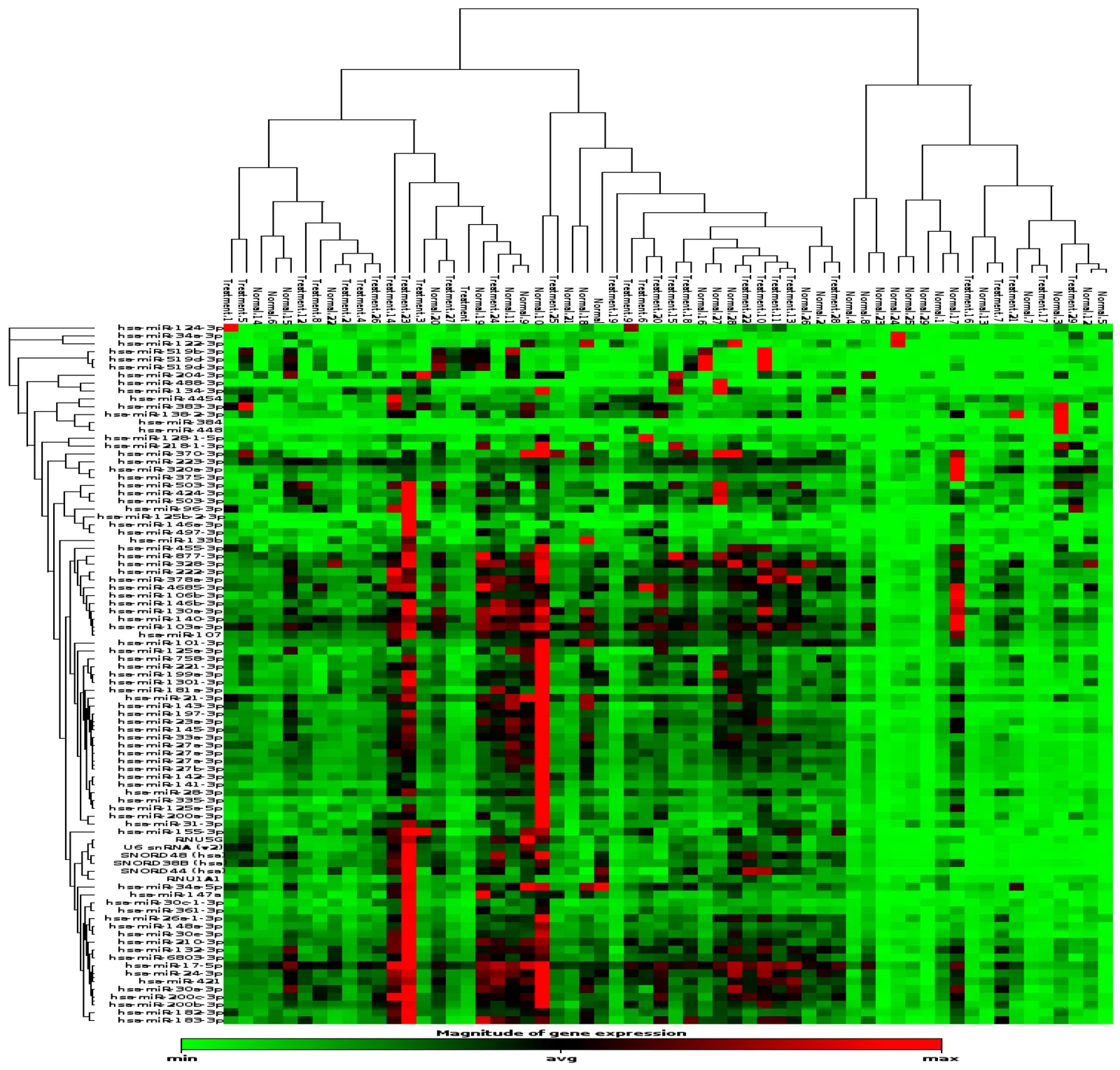
Changes in miRNA expression.

The target genes and associated signaling pathways were compiled from previously reported experimental and bioinformatic studies. These targets were not experimentally validated in the present study but were evaluated to interpret the potential obesity-related biological effects of the corresponding miRNAs (Table 5, Figure 1).

## 3. Discussion

In the present study, hsa-miR-155-3p expression was increased in individuals with obesity, whereas hsa-miR-223-3p and hsa-miR-199a-3p expression levels were decreased. Moreover, the significantly higher lymphocyte count observed in the obese group suggests that these miRNA alterations may be linked to immune system activation. These findings are consistent with previous studies indicating that obesity is not merely a metabolic disorder but also a chronic inflammatory condition in which the immune system is actively involved [14,15,16].

MicroRNAs (miRNAs) are key epigenetic regulators that control gene expression at the post-transcriptional level and are involved in fundamental biological processes such as inflammation, metabolism, and immune response [17]. Because a single miRNA can regulate multiple target genes, alterations in miRNA expression may exert broad biological effects in complex diseases such as obesity.

The increased expression of miR-155-3p observed in our study supports the central role of this microRNA in inflammatory processes. Previous studies have shown that miR-155 activates inflammatory signaling pathways by targeting genes such as SOCS1 and SHIP1 and enhances NF-κB-mediated responses [25,35]. In addition, miR-155 has been reported to play a regulatory role in T lymphocyte activation and macrophage functions [25,35]. In this context, the increase in miR-155 expression observed in individuals with obesity, together with the elevated lymphocyte count, suggests that immune system activation is supported at the molecular level.

In contrast, the decreased expression of miR-223-3p indicates a potential impairment in the regulation of inflammation. miR-223 is known to suppress inflammatory responses through targets such as the NLRP3 inflammasome and STAT3 [36,37]. In addition, this microRNA has been shown to contribute to the control of inflammation by regulating myeloid cell functions [36]. Therefore, the decrease in miR-223-3p observed in our study suggests that the inflammatory process may not be adequately suppressed in individuals with obesity.

The decreased expression of miR-199a-3p may indicate that hypoxia- and metabolic stress-related mechanisms are affected in obesity. miR-199a has been reported to regulate cellular stress responses through the HIF1A and mTOR pathways and to play a role in adaptation to hypoxia [38,39]. In addition, this microRNA has been shown to be associated with metabolic regulation and adipocyte function [39]. In this context, the observed decrease may be related to the hypoxic microenvironment that develops in adipose tissue during obesity.

In the present study, significant differences were detected in the expression levels of certain miRNAs in comparisons performed according to BMI and triglyceride levels. Previous studies have reported that miR-143 is involved in adipogenesis through the ERK5 and AKT pathways [41], whereas miR-375 affects the insulin signaling pathway through PDK1 and JAK2 [42]. Similarly, miR-122 is known to regulate FASN and SREBP1, which are involved in lipid synthesis [43]. These findings suggest that miRNAs may influence lipid metabolism and energy homeostasis in obesity.

When the obtained data are evaluated together, inflammation, lipid metabolism, and cellular stress responses appear to be interconnectedly affected in obesity. In particular, the increase in miR-155 suggests activation of the inflammatory response, whereas the decreases in miR-223 and miR-199a indicate a potential impairment in the regulation of this process. This finding supports the concept that obesity is a complex disease affecting multiple biological systems.

Lymphocytes are important cellular components of chronic low-grade inflammation that develops in obesity. In the present study, the increased lymphocyte count observed in individuals with obesity, together with alterations in miRNA expression, may contribute to a better understanding of immune–metabolic interactions. Previous studies have shown that lymphocyte-derived miRNAs play important regulatory roles in immune responses [44]. Although hematological parameters have previously been investigated in obese and pre-obese individuals, studies evaluating the relationship between lymphocyte levels and miRNA expression remain limited [45]. Obesity-associated microRNA dysregulation, lymphocyte-related inflammation, and metabolic impairment appear to represent interconnected biological processes. Recent studies published in 2024 demonstrated that adipose tissue is a major source of circulating microRNAs and that obesity is characterized by widespread dysregulation of adipose-derived miRNAs. Among these, increased expression of miR-802 has been shown to promote macrophage infiltration, amplify adipose tissue inflammation, and exacerbate systemic insulin resistance, further supporting the concept that adipose tissue actively contributes to obesity-associated immune dysfunction rather than serving merely as an energy storage organ [46,47].

Evidence from studies published in 2025 further strengthened these observations in human populations. Circulating microRNA panels demonstrated promising diagnostic potential for distinguishing obesity-associated metabolic dysfunction. Specifically, increased miR-155 expression was associated with enhanced inflammatory activity and impaired glucose metabolism, whereas altered expression of miR-142 and miR-378 was linked to differences in metabolic risk profiles. Furthermore, serum and visceral adipose tissue expression of miR-450a-5p showed significant positive correlations with fasting plasma glucose, triglycerides, total cholesterol, and LDL-C, suggesting that circulating miRNAs may serve as integrated biomarkers reflecting both inflammatory and metabolic disturbances in obesity [48,49,50]. Interestingly, recent clinical investigations also demonstrated an inverse association between peripheral lymphocyte counts and the likelihood of metabolically healthy obesity. In addition, several cohort studies reported that elevated lymphocyte counts frequently coexisted with increased serum cholesterol and triglyceride concentrations. These findings support the concept that alterations in immune cell composition are not merely secondary phenomena but are closely linked to the metabolic phenotype observed in obesity. Our findings are consistent with this emerging concept, although it should be emphasized that the present study demonstrates an association rather than a causal relationship between lymphocyte counts and circulating microRNA expression [51].

More recently, studies published in 2026 suggested that visceral adipose tissue microRNA profiles are primarily shaped by obesity itself rather than by diabetes. Integrated miRNA–mRNA network analyses revealed significant interactions with inflammatory pathways, lipid metabolism, and insulin signaling. Furthermore, several investigators proposed that combining circulating microRNAs with established metabolic biomarkers such as HOMA-IR, C-reactive protein (CRP), and adiponectin could substantially improve metabolic risk stratification compared with the use of single biomarkers alone [52,53,54]. Our analytical approach is also consistent with previous studies in which peripheral blood samples were collected and the expression profiles of predefined circulating miRNAs (including panels of 89 miRNAs) were evaluated using comparable high-throughput analytical workflows. These methodological similarities support the validity and reproducibility of the approach used in the present study [55]. These findings provide additional biological support for the interpretation of our results and highlight the potential value of circulating microRNAs as complementary biomarkers reflecting the complex interaction between immune regulation and metabolic dysfunction in obesity.

In this respect, the present study provides a distinctive approach by jointly assessing lymphocyte parameters and miRNA expression, thereby contributing to the understanding of interactions between immune cells and epigenetic regulators in obesity.

However, this study has several limitations. First, the relationships between miRNA expression and clinical parameters were not evaluated at the individual level using correlation analysis. In addition, the lack of correction for multiple comparisons requires that the findings be interpreted with caution. Further studies with larger sample sizes and functional validation are needed to confirm these findings. Although participants with diabetes mellitus, hypothyroidism, and other predefined major comorbidities were excluded, the study did not include an exhaustive etiological or metabolic stratification of obesity. Therefore, residual clinical heterogeneity within the obese group cannot be completely excluded. Future studies with larger cohorts and detailed metabolic and endocrine phenotyping are required to confirm whether the observed miRNA alterations are specifically attributable to obesity. We recognize that the correlation analysis and the application of BMI—which provides an incomplete assessment of body composition and fat distribution—constitute limitations of the present work. To achieve a more robust evaluation, subsequent studies should integrate precise measures of body composition and adipose tissue quality.

## 4. Materials and Methods

### 4.1. Ethical Approval

This study was conducted in accordance with the principles of the Declaration of Helsinki of the World Medical Association. The study protocol was approved by the Fırat University Non-Interventional Clinical Research Ethics Committee (08.04.2026-46549). Written informed consent was obtained from all participants.

### 4.2. Sample and Study Groups

The study included 50 individuals with obesity and 25 age- and sex-matched healthy controls, aged 18–69 years. Participants with obesity were recruited from the Diabetes Unit of the Department of General Surgery, Faculty of Medicine, Fırat University. This specialized outpatient unit provides routine metabolic evaluation and follow-up for individuals with obesity, irrespective of whether bariatric surgery is indicated. Recruitment from this unit did not imply a diagnosis of diabetes mellitus. Eligible participants were adults with a body mass index (BMI) of ≥30 kg/m^2^ who attended the unit for routine metabolic assessment. Blood samples were collected during outpatient evaluation before any medical or surgical intervention. Eligibility was determined based on medical history, clinical examination, and available routine metabolic assessments. Individuals with diabetes mellitus, hypothyroidism, acute or chronic infections, autoimmune or inflammatory diseases, malignancy, chronic liver or kidney disease, hematological disorders, pregnancy, recent surgery or trauma, or alcohol abuse were excluded. Participants receiving corticosteroids, immunosuppressive drugs, anti-inflammatory agents, or lipid-lowering medications were also excluded. In addition, individuals who had previously undergone bariatric surgery or another surgical intervention before blood collection, as well as those showing evidence of active infection or inflammation at enrollment, were not included. The control group consisted of 25 age- and sex-matched healthy volunteers recruited during routine health examinations. Control participants had no history of obesity, diabetes mellitus, hypertension, cardiovascular disease, autoimmune disease, malignancy, acute or chronic inflammatory disease, or active infection and were not receiving regular medication. All participants underwent clinical evaluation before enrollment, and fasting venous blood samples were collected after an overnight fast.

### 4.3. Biochemical and Hematological Analyses

Fasting venous blood samples were collected from all participants after an overnight fast. Blood samples were used for both biochemical and hematological analyses. Samples collected for serum separation were centrifuged and stored at −20 °C until analysis. Serum total cholesterol (TC), high-density lipoprotein cholesterol (HDL-C), low-density lipoprotein cholesterol (LDL-C), and triglyceride (TG) concentrations were determined using an automated clinical chemistry analyzer (Siemens Healthineers AG, Erlangen, Germany). Hematological parameters, including hemoglobin, neutrophil, lymphocyte, and platelet counts, were measured using a Sysmex XN-1000 SA-01 hematology analyzer (Sysmex Corporation, Kobe, Japan).

### 4.4. RNA Isolation and miRNA Expression Analysis

RNA stabilization reagent was added to blood samples collected into EDTA-containing tubes, and the samples were stored at −20 °C until analysis. Total RNA isolation was performed using the Zymo Total RNA isolation kit (Zymo Research, Irvine, CA, USA) according to the manufacturer’s protocol. cDNA was synthesized from the isolated RNA samples, and miRNA expression analysis was performed using the Fluidigm 96.96 Dynamic Array IFC and BioMark RT-PCR system (Fluidigm Corporation, South San Francisco, CA, USA). Using this system, 86 miRNAs and 7 reference genes/endogenous controls were analyzed in a total of 75 samples. miRNA expression levels were calculated based on Ct values using the ΔCt method.

For normalization, the reference genes/endogenous controls included in the panel were evaluated, and among 5S rRNA, SNORD44, and U6 snRNA, 5S rRNA was identified as the most stable endogenous control. Therefore, normalization in miRNA expression analyses was performed using 5S rRNA.

### 4.5. Statistical Analysis

The sample size of the study was calculated using G*Power software (version 3.1, Heinrich Heine University, Düsseldorf, Germany), with a statistical power of 85% and a type I error rate of 0.05, resulting in 50 participants in the obese group and 25 participants in the control group. Statistical analyses were performed using SPSS for Windows, version 15.0. Data were expressed as mean ± standard deviation (mean ± SD). The normality of data distribution was assessed using the Shapiro–Wilk test. Comparisons between groups were performed using the independent samples Student’s *t*-test. miRNA expression analyses, including ΔCt calculation, fold regulation (2^−ΔΔCt^) and statistical evaluation, were performed using the Qiagen RNA Portal Data Analysis platform (https://rnaportal.qiagen.com/rnaportalui/#/ap/projects, accessed on 23 October 2024). A *p* value of <0.05 was considered statistically significant.

All analyses were performed using 5S rRNA as the housekeeping gene for normalization, and the Ct cut-off value was set at 30. miRNA expression levels were calculated using the 2^−ΔΔCt^ method. Fold-change values were determined as the ratio of normalized miRNA expression in the test group to that in the control group. Fold-regulation values were calculated to indicate the direction of fold-change results: values greater than 1 indicated upregulation, whereas values less than 1 indicated downregulation. For statistical analyses, a parametric, unpaired, two-tailed Student’s *t*-test was used for between-group comparisons, and a *p* value of <0.05 was considered statistically significant.

## 5. Conclusions

In this study, miR-155-3p expression was increased in individuals with obesity, whereas miR-223-3p and miR-199a-3p expression levels were decreased. Individuals with obesity also exhibited higher peripheral blood lymphocyte counts, and additional differences in miRNA expression were observed in analyses based on BMI and triglyceride levels. Collectively, these findings demonstrate that alterations in whole-blood miRNA expression are associated with hematological and metabolic changes in obesity. Because miRNA expression was assessed in whole-blood samples rather than in isolated lymphocyte populations, the observed alterations cannot be interpreted as lymphocyte-specific or as evidence that lymphocytes were the cellular source of the differentially expressed miRNAs. Instead, the results indicate associations among circulating miRNA profiles, peripheral blood lymphocyte counts, BMI, and lipid-related metabolic parameters. The identified miRNAs may represent potential biomarker candidates for obesity-associated metabolic and inflammatory alterations. However, studies involving larger and clinically well-characterized cohorts, isolated cell populations, functional validation, and appropriate correction for multiple comparisons are required to clarify their cellular origins, biological significance, and clinical applicability.

## Figures and Tables

**Table 1:** Demographic and laboratory findings of the obese and control groups.

	Healthy	Obese	*p*-Value
Gender (Male/Female)	13/12 (n = 25)	24/26 (n = 50)	0.744
Age, years	38.92 ± 14.70 37.00 (22.00–69.00)	39.70 ± 14.18 37.00 (18.00–69.00)	0.8275
Body weight, kg	62.92 ± 9.17 60.0 (48.0–78.0)	98.08 ± 22.39 95.0 (65.0–150.0)	<0.0001
Height, cm	170.24 ± 11.46 170.0 (150.0–189.0)	169.52 ± 9.71 168.0 (153.0–187.0)	0.7765
BMI, kg/m^2^	21.62 ± 1.38 21.90 (18.90–23.60)	34.11 ± 7.19 29.70 (24.8–50.80)	<0.0001
HDL-C, mmol/L	1.142 ± 0.364 1.142 (0.544–1.691)	1.024 ± 0.347 1.247 (0.492–2.305)	0.1756
LDL-C, mmol/L	2.611 ± 0.6241 2.538 (1.595–4.299)	3.057 ± 0.869 1.142 (1.217–5.175)	0.0253
CHOL, mmol/L	3.501 ± 0.911 3.522 (1.994–5.957)	4.611 ± 0.945 4.442 (3.134–6.605)	<0.0001
Triglyceride, mmol/L	1.448 ± 0.748 1.311 (0.667–4.283)	2.611 ± 0.945 1.882 (0.633–5.876)	0.0144
Hemoglobin, mmol/L	8.32 ± 1.73 8.25 (5.96–10.36)	8.46 ± 1.06 8.50 (6.33–10.80)	0.6418
Neutrophil, µL	5016.80 ± 2430.33 4600 (1420–10,750)	5227.60 ± 2356.56 4960 (1710–12,780)	0.7188
Lymphocyte	2161.60 ± 1257.89 2060 (410–6910)	2772.80 ± 1033.06 2480 (1120–5420)	0.0279
Monocyte	613.20 ± 234.44 555 (310–1360)	614.98 ± 232.90 555 (210–1370)	0.9752
Platelet	288,488.0 ± 134,603.0 255,000 (21,200–640,000)	272,400.0 ± 81,387.5 266,500 (144,000–524,000)	0.5216

**Table 2:** Differentially expressed miRNAs associated with lymphocyte levels.

miRNA ID	Fold Regulation	*p*-Value
hsa-miR-223-3p	−2.47	0.004527
hsa-miR-199a-3p	−2.76	0.006351
hsa-miR-155-3p	2.48	0.048461

**Table 3:** Significant miRNA alterations associated with body mass index (BMI).

miRNA ID	Fold Regulation	*p*-Value
hsa-miR-199a-3p	−2.03	0.032176
hsa-miR-143-3p	−2.83	0.011588

**Table 4:** Differentially expressed miRNAs associated with triglyceride levels.

miRNA ID	Fold Regulation	*p*-Value
hsa-miR-424-3p	−2.03	0.041868
hsa-miR-375-3p	−2.23	0.030207

**Table 5:** Potential targets, biological functions, and signaling pathways associated with differentially expressed miRNAs.

miRNA	Target Gene(s)	Biological Function	Associated Signaling Pathway
miR-155-3p	*SOCS1*, *SHIP1*, *TAB2*	Increased inflammation and cytokine signaling	NF-κB, JAK/STAT [32,33,34]
miR-223-3p	*NLRP3*, *STAT3*	Suppression of inflammatory response	Inflammatory response [35,36]
miR-199a-3p	*HIF1A*, *mTOR*	Regulation of hypoxic response and metabolic stress	HIF-1, mTOR [37,38]
miR-146a-3p	*IRAK1*, *TRAF6*	Immune regulation via negative feedback	Toll-like receptor signaling [39,40]
miR-143-3p	*ERK5*, *AKT*	Regulation of adipogenesis and cell growth	MAPK, PI3K/Akt [41]
miR-375-3p	*PDK1*, *JAK2*	Regulation of glucose and lipid metabolism	Insulin signaling pathway [42]
miR-122-3p	*FASN*, *SREBP1*	Lipid synthesis and fatty acid metabolism	Lipogenesis [43]
miR-204-3p	*BCL2*, *SIRT1*	Apoptosis and cellular stress response	Cellular stress response [44]
miR-424-3p	*CCND1*, *VEGFA*	Cell cycle and energy balance	Cell proliferation [45]

## Data Availability

The datasets generated and analyzed during the current study are available from the corresponding author upon reasonable request.

## References

[1] World Health Organization (2024). Obesity and Overweight.

[2] Lustig R.H., Collier D., Kassotis C., Roepke T.A., Kim M.J., Blanc E., Barouki R., Bansal A., Cave M.C., Chatterjee S. (2022). Obesity I: Overview and molecular and biochemical mechanisms. Biochem. Pharmacol..

[3] Miller J. (2023). Genetic Obesity-Causes and Treatments. Pediatr. Ann..

[4] Thaker V. (2017). V Genetic and Epigenetic Causes of Obesity. Adolesc. Med. State Art Rev..

[5] Mahmoud R., Kimonis V., Butler M.G. (2022). Genetics of Obesity in Humans: A Clinical Review. Int. J. Mol. Sci..

[6] Baxter J., Armijo P.R., Flores L., Krause C., Samreen S., Tanner T. (2019). Updates on Monogenic Obesity in a Multifactorial Disease. Obes. Surg..

[7] Carvalho L.M.L., D’Angelo C.S., Villela D., da Costa S.S., de Lima Jorge A.A., da Silva I.T., de Oliveira Scliar M., Chaves L.D., Krepischi A.C.V., Koiffmann C.P. (2022). Genetic Investigation of Syndromic Forms of Obesity. Int. J. Obes..

[8] Loos R.J.F., Yeo G.S.H. (2022). The Genetics of Obesity: From Discovery to Biology. Nat. Rev. Genet..

[9] Oussaada S.M., van Galen K.A., Cooiman M.I., Kleinendorst L., Hazebroek E.J., van Haelst M.M., ter Horst K.W., Serlie M.J. (2019). The Pathogenesis of Obesity. Metabolism.

[10] Wu F.Y., Yin R.X. (2022). Recent Progress in Epigenetics of Obesity. Diabetol. Metab. Syndr..

[11] Zhong H., Ma M., Liang T., Guo L. (2018). Role of MicroRNAs in Obesity-Induced Metabolic Disorder and Immune Response. J. Immunol. Res..

[12] Bartel D.P. (2004). MicroRNAs: Genomics, biogenesis, mechanism, and function. Cell.

[13] Rakib A., Kiran S., Mandal M., Singh U.P. (2022). MicroRNAs: A crossroad that connects obesity to immunity and aging. Immun. Ageing.

[14] Hotamisligil G.S. (2006). Inflammation and metabolic disorders. Nature.

[15] O’Rourke R.W., White A.E., Metcalf M.D., Olivan A.S., Mitra P., Larison W.G., Cheang E.C., Viggiano A.L., Marks D.L. (2013). Systemic inflammation and immune cell infiltration in obesity. J. Clin. Endocrinol. Metab..

[16] Winer S., Chan Y., Paltser G., Truong D., Tsui H., Bahrami J., Dorfman R., Wang Y., Zielenski J., Mastronardi F. (2009). Normalizing obesity-associated insulin resistance through immunotherapy. Nat. Med..

[17] Seyhan A.A. (2024). Trials and Tribulations of MicroRNA Therapeutics. Int. J. Mol. Sci..

[18] De Guire V., Robitaille R., Tetreault N., Guerin R., Menard C., Bambace N., Sapieha P. (2013). Circulating miRNAs as sensitive and specific biomarkers for the diagnosis and monitoring of human diseases: Promises and challenges. Clin. Biochem..

[19] Condrat C.E., Thompson D.C., Barbu M.G., Bugnar O.L., Boboc A., Cretoiu D., Suciu N., Cretoiu S.M., Voinea S.C. (2020). miRNAs as Biomarkers in Disease: Latest Findings Regarding Their Role in Diagnosis and Prognosis. Cells.

[20] Gareev I., de Jesus Encarnacion Ramirez M., Goncharov E., Ivliev D., Shumadalova A., Ilyasova T., Wang C. (2023). MiRNAs and lncRNAs in the regulation of innate immune signaling. Non-Coding RNA Res..

[21] Nunez Lopez Y.O., Coen P.M., Goodpaster B.H., Seyhan A.A. (2017). Gastric bypass surgery with exercise alters plasma microRNAs that predict improvements in cardiometabolic risk. Int. J. Obes..

[22] Nunez Lopez Y.O., Garufi G., Pasarica M., Seyhan A.A. (2018). Elevated and Correlated Expressions of miR-24, miR-30d, miR-146a, and SFRP-4 in Human Abdominal Adipose Tissue Play a Role in Adiposity and Insulin Resistance. Int. J. Endocrinol..

[23] Nunez Lopez Y.O., Garufi G., Seyhan A.A. (2016). Altered levels of circulating cytokines and microRNAs in lean and obese individuals with prediabetes and type 2 diabetes. Mol. Biosyst..

[24] Rayner K.J., Suárez Y., Dávalos A., Parathath S., Fitzgerald M.L., Tamehiro N., Fisher E.A., Moore K.J., Fernández-Hernando C. (2010). MiR-33 contributes to the regulation of cholesterol homeostasis. Science.

[25] O’Connell R.M., Rao D.S., Baltimore D. (2012). microRNA regulation of inflammatory responses. Annu. Rev. Immunol..

[26] Arner E., Mejhert N., Kulyté A., Balwierz P.J., Pachkov M., Cormont M., Lorente-Cebrián S., Ehrlund A., Laurencikiene J., Hedén P. (2012). Adipose tissue microRNAs as regulators of CCL2 production in human obesity. Diabetes.

[27] Abu-Farha M., Cherian P., Al-Khairi I., Nizam R., Alkandari A., Arefanian H., Tuomilehto J., Al-Mulla F., Abubaker J. (2019). Reduced MiR-181d Level in Obesity and Its Role in Lipid Metabolism via Regulation of ANGPTL3. Sci. Rep..

[28] Liu Y., Liu H., Li Y., Mao R., Yang H., Zhang Y., Zhang Y., Guo P., Zhan D., Zhang T. (2020). Circular RNA SAMD4A Controls Adipogenesis in Obesity through the MiR-138-5p/EZH2 Axis. Theranostics.

[29] Liu J., Wang H., Zeng D., Xiong J., Luo J., Chen X., Chen T., Xi Q., Sun J., Ren X. (2023). The Novel Importance of MiR-143 in Obesity Regulation. Int. J. Obes..

[30] Tsitsiou E., Lindsay M.A. (2009). microRNAs and the immune response. Curr. Opin. Pharmacol..

[31] Valadi H., Ekström K., Bossios A., Sjöstrand M., Lee J.J., Lötvall J.O. (2007). Exosome-mediated transfer of mRNAs and microRNAs is a novel mechanism of genetic exchange between cells. Nat. Cell Biol..

[32] Zhang L., Hou D., Chen X., Li D., Zhu L., Zhang Y., Li J., Bian Z., Liang X., Cai X. (2012). Exogenous plant MIR168a specifically targets mammalian LDLRAP1: Evidence of cross-kingdom regulation by microRNA. Cell Res..

[33] Witwer K.W. (2012). XenomiRs and miRNA homeostasis in health and disease: Evidence that diet and dietary miRNAs directly and indirectly influence circulating miRNA profiles. RNA Biol..

[34] Karkeni E., Astier J., Tourniaire F., El Abed M., Romier B., Gouranton E., Wan L., Borel P., Salles J., Walrand S. (2016). Obesity-associated Inflammation Induces microRNA-155 Expression in Adipocytes and Adipose Tissue: Outcome on Adipocyte Function. J. Clin. Endocrinol. Metab..

[35] Johnnidis J.B., Harris M.H., Wheeler R.T., Stehling-Sun S., Lam M.H., Kirak O., Brummelkamp T.R., Fleming M.D., Camargo F.D. (2008). Regulation of progenitor cell proliferation and granulocyte function by microRNA-223. Nature.

[36] Bauernfeind F., Rieger A., Schildberg F.A., Knolle P.A., Schmid-Burgk J.L., Hornung V. (2012). NLRP3 inflammasome activity is negatively controlled by miR-223. J. Immunol..

[37] Rane S., He M., Sayed D., Vashistha H., Malhotra A., Sadoshima J., Vatner D.E., Vatner S.F., Abdellatif M. (2009). Downregulation of miR-199a derepresses hypoxia-inducible factor-1alpha and Sirtuin 1 and recapitulates hypoxia preconditioning in cardiac myocytes. Circ. Res..

[38] Du H., Zhao Y., Li H., Wang D.W., Chen C. (2021). Roles of MicroRNAs in Glucose and Lipid Metabolism in the Heart. Front. Cardiovasc. Med..

[39] Taganov K.D., Boldin M.P., Chang K.J., Baltimore D. (2006). NF-kappaB-dependent induction of microRNA miR-146, an inhibitor targeted to signaling proteins of innate immune responses. Proc. Natl. Acad. Sci. USA.

[40] Nahid M.A., Satoh M., Chan E.K. (2015). Interleukin 1β-Responsive MicroRNA-146a Is Critical for the Cytokine-Induced Tolerance and Cross-Tolerance to Toll-Like Receptor Ligands. J. Innate Immun..

[41] Esau C., Kang X., Peralta E., Hanson E., Marcusson E.G., Ravichandran L.V., Sun Y., Koo S., Perera R.J., Jain R. (2004). MicroRNA-143 regulates adipocyte differentiation. J. Biol. Chem..

[42] Poy M.N., Eliasson L., Krutzfeldt J., Kuwajima S., Ma X., Macdonald P.E., Pfeffer S., Tuschl T., Rajewsky N., Rorsman P. (2004). A pancreatic islet-specific microRNA regulates insulin secretion. Nature.

[43] Yu J., Peng J., Luan Z., Zheng F., Su W. (2019). MicroRNAs as a Novel Tool in the Diagnosis of Liver Lipid Dysregulation and Fatty Liver Disease. Molecules.

[44] Haralambieva I.H., Ratishvili T., Goergen K.M., Grill D.E., Simon W.L., Chen J., Ovsyannikova I.G., Poland G.A., Kennedy R.B. (2025). Effect of lymphocyte miRNA expression on influenza vaccine-induced immunity. Vaccine.

[45] Kargün K., Aygen E., Ebiloğlu M.F., Alayunt N.Ö., Dalkılıç L.K. (2025). Evaluation of miRNA Profile and Its Relationship with Metabolic Disorders in Obese and Pre-Obese Patients. Curr. Issues Mol. Biol..

[46] Liu X., Sun H., Zheng L., Zhang J., Su H., Li B., Wu Q., Liu Y., Xu Y., Song X. (2024). Adipose-derived miRNAs as potential biomarkers for predicting adulthood obesity and its complications: A systematic review and bioinformatic analysis. Obes. Rev..

[47] Yang Y., Huang B., Qin Y., Wang D., Jin Y., Su L., Wang Q., Pan Y., Zhang Y., Shen Y. (2024). Adipocyte microRNA-802 promotes adipose tissue inflammation and insulin resistance by modulating macrophages in obesity. eLife.

[48] Villagrán-Silva F., Loren P., Sandoval C., Lanas F., Salazar L.A. (2025). Circulating microRNAs as Potential Biomarkers of Overweight and Obesity in Adults: A Narrative Review. Genes.

[49] Markina N.O., Matveev G.A., Zasypkina K.A., Khromova N.V., Babenko A.Y., Shlyakhto E.V. (2025). Comparative Analysis of Gene and MicroRNA Expression in Subcutaneous Adipose Tissue in Metabolically Healthy and Unhealthy Obesity. Int. J. Mol. Sci..

[50] Zhu J., Hou Y., Yu W., Wang J., Chu X., Zhang X., Pang H., Ma D., Tang Y., Li M. (2025). Adipose tissue-derived microRNA-450a-5p induces type 2 diabetes mellitus by downregulating DUSP10. Mol. Biomed..

[51] Li Z., Yao Z., Liu Q. (2025). The association between white blood cell counts and metabolic health obesity among US adults. Front. Nutr..

[52] Villa-Fernández E., García A.V., Gallardo-Nuell L., García-Villarino M., Fernández-García J., Martin Alonso A., Lozano-Aida C., Suárez Gutiérrez L., Pujante P., Ares J. (2026). Integrated miRNA-mRNA Analysis Reveals Obesity-Driven Regulatory Networks in Human Visceral Adipose Tissue with and Without Type 2 Diabetes. Diabetes Obes. Metab..

[53] Di Maio G., Tafuri M.G., Casillo M., Messina A., Allocca S., Villano I., Moscatelli F., Monda A., La Marra M., Monda V. (2026). Physiological Regulation of Nutritional and Metabolic Biomarkers in Obesity: Implications for Precision Nutrition. Nutrients.

[54] Podraza J., Wąsowski M., Jonas M.I., Lisik W., Jonas M., Binda A., Jaworski P., Tarnowski W., Noszczyk B., Puzianowska-Kuźnicka M. (2026). Negative correlation between microRNA and insulin sensitivity gene expression in adipose tissue: Potential implications for diabetes and neurodegenerative diseases. Nutr. Metab..

[55] Pektaş S.D., Kara M., Doğan G., Pektaş M.B., Baloğlu M.C., Sadi G. (2022). Differential Expression and in Silico Functional Analysis of Plasma MicroRNAs in the Pathogenesis of Non-segmental Vitiligo. Indian J. Dermatol..

